# Heterologous expression and functional characterization of recombinant arenin to assess its anticancer and wound-healing potential

**DOI:** 10.1186/s40643-025-00986-2

**Published:** 2025-12-06

**Authors:** Enrique Hidalgo-Vázquez, Jesús Hernández-Pérez, Marilena Antunes-Ricardo, Calef Sánchez-Trasviña, Mario E. Barocio, María Isabela Avila Rodríguez, Jorge Benavides

**Affiliations:** 1 Tecnologico de Monterrey, School of Engineering and Science, Ave. Eugenio Garza Sada Sur 2501, 64849 Monterrey, N.L. Mexico; 2 Tecnologico de Monterrey, Institute for Obesity Research, Ave. Eugenio Garza Sada Sur 2501, 64849 Monterrey, N.L. Mexico; 3 Present Address: Tecnologico de Monterrey, Centro de Primera Infancia, Ave. Eugenio Garza Sada Sur 2501, 64849 Monterrey, N.L. Mexico

**Keywords:** Arenin, Amphibian, Kunitz-type peptide, Wound healing, Anticancer activity

## Abstract

Arenin is a cystine-rich Kunitz-type protease inhibitor originally isolated from the skin secretion of the tree frog *Dryophytes arenicolor*, whose limited natural yield has hindered comprehensive functional studies. In this work, we established an efficient recombinant production system in *Escherichia coli* and evaluated its anticancer and wound-healing properties. A codon-optimized arenin gene, fused to an N-terminal 6 × His-TEV tag, was cloned into a T7-lac expression cassette. IPTG induction at 30 °C and 37 °C revealed distinct temperature-dependent partitioning: at 30 °C, arenin predominantly accumulated in the soluble fraction, whereas at 37 °C, it was confined to inclusion bodies. Both strategies yielded ≥ 90% pure peptide, producing 7.8 ± 0.6 mg and 12.4 ± 1.0 mg per 250 mL culture, respectively. Bioassays showed that HDFa fibroblasts tolerated 31.25–500 µg mL^−1^ arenin and had increased viability at 1000 µg mL^−1^. ER⁺ MCF-7 cells showed mild inhibition at low doses but growth stimulation at the highest, suggesting hormetic protease–receptor effects. Caco-2 cells were sensitive, with viability at 60.2 ± 3.4% at 31.25 µg mL^−1^ and below 80% up to 250 µg mL^−1^. Scratch-wound assays under serum deprivation or high glucose showed complete closure within 72 h at all arenin concentrations, comparable to *Centella asiatica* (10 µg mL^−1^), consistent with other Kunitz-family peptide activities in diverse protease environments. Overall, our results demonstrate the feasibility of a heterologous expression strategy for large-scale arenin production and highlight its dual functional potential, pro-regenerative effects in fibroblasts, and selective cytotoxicity toward hormone-independent cancer cells.

**Graphical abstract:**

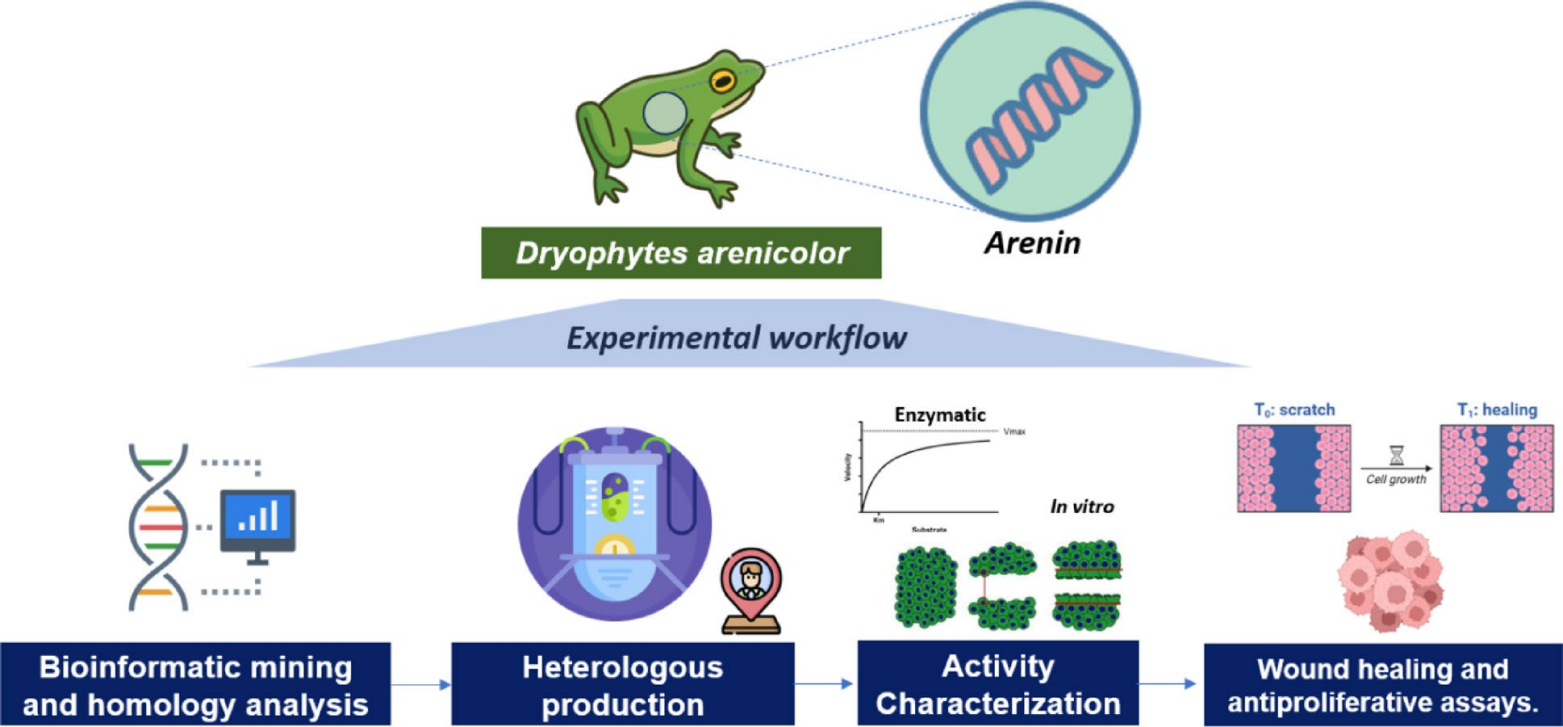

## Introduction

Many cultures have long recognized anurans as valuable sources of natural remedies and bioactive compounds, incorporating them into traditional pharmacopeias for their healing and protective properties. Much of this biological versatility stems from the specialized granular glands in amphibian skin, which secrete complex cocktails of peptides, proteins, alkaloids, steroids, and biogenic amines. These secretions not only serve as critical defenses against microbial invasion and predation but also support key physiological processes such as wound healing and tissue regeneration (König et al. 2015; Xu and Lai 2015). Within this rich array of molecules, short cystine‐rich peptides stand out for their potent antimicrobial, antioxidant, anticancer, and pro-regenerative activities, all housed within a remarkably stable disulfide‐bonded framework (Cao et al. 2018; Huang et al. 2017).

Among these, Kunitz‐type peptides form a particularly well‐defined subclass. Comprised of roughly 60 amino acids and stabilized by three characteristic disulfide bridges, Kunitz inhibitors bind serine proteases with high affinity yet fully reversible kinetics. These unique inhibitory properties have positioned Kunitz‐type peptides as promising therapeutic leads for applications ranging from inflammation control to cancer treatment and tissue repair (Chen et al. 2016; Fratini et al. 2022; Ranasinghe et al. 2015; Rashno et al. 2023; Shigetomi et al. 2010).

The anuran family *Hylidae*, particularly the genus *Dryophytes*, has emerged as an exceptionally abundant source of these bioactive molecules. Ethnopharmacological studies conducted in central Mexico have documented traditional topical applications of the canyon tree frog, *Dryophytes arenicolor,* for persistent dermatological conditions, suggesting the presence of potent bioactive compounds in its skin secretions (Alonso-Castro et al. 2011). Recent proteotranscriptomic investigations have validated this hypothesis, identifying and characterizing arenin, a novel and predominant Kunitz-type inhibitor present in the secretions of this species, which demonstrates potential in vitro inhibition of trypsin-like proteases. Preliminary bioactivity screenings have also indicated the anticancer and antimicrobial capabilities of arenin (Hernández-Pérez et al. 2018). However, further characterization has been significantly limited by the minimal quantities extractable from natural sources.

Heterologous expression using *Escherichia coli* presents a viable strategy for obtaining the requisite milligram-scale quantities necessary for in-depth biochemical and cellular analyses. Nonetheless, cystine-rich peptides frequently aggregate into misfolded inclusion bodies during bacterial expression, necessitating precisely optimized refolding protocols to restore their native structure and bioactivity (de Marco 2009). In this study, we address these critical technical challenges, developing an efficient bioprocess to produce recombinant arenin using *E. coli* as an expression system, coupled with a streamlined affinity-based purification and refolding procedure. Furthermore, we characterize the functionality of the expressed recombinant arenin in terms of its serine protease inhibitory activity, an intrinsic characteristic of arenin and other Kunitz-type peptides, as an indirect approach to validate its folding state. Moreover, in vitro antitumor and wound-healing functional characterization of the recombinant arenin was conducted, underscoring its potential as a versatile therapeutic candidate that bridges oncology and regenerative medicine.

## Materials and methods

### Reagents

Isopropyl β-D-Thiogalactopyranoside (IPTG), sulfuric acid, rubidium chloride, glycerol 99.5%, imidazole, sodium phosphate, sodium chloride, potassium chloride, urea ≥ 99%, Tween 20, non-fat skim milk powder, recombinant porcine trypsin, Nα-benzoyl-L-arginine ethyl ester (BAEE), and 8 M urea were obtained from Sigma Aldrich (St. Louis, MO, USA).

Coomassie Brilliant Blue G-250, Precision Plus Protein™ Dual Xtra, tricine, tris, 12% Tris–Glycine precast gels, and Clarity™ Western ECL substrate were acquired from Bio-Rad (Hercules, CA, USA). Pierce Quantitative Colorimetric Peptide Assay Kit, Dulbecco's Modified Eagle Medium. Nutrient Mixture F12 (DMEM-F12, powder), penicillin–streptomycin, phosphate-buffered saline (PBS) (PubChem CID: 24978514), SOC medium, and Pierce™ BCA (Bicinchoninic Acid assay) Protein Assay Kit were purchased from Thermo Fisher Scientific Inc. (Waltham, MA, USA). CellTiter 96® AQueous One Solution Cell Proliferation Assay and Griess Reagent System were obtained from Promega Corporation (Madison, WI, USA). HisTrap HP 1 mL column was purchased from Cytiva Life Sciences (Marlborough, MA, USA). Absolute ethanol (PubChem CID: 702) was obtained from Desarrollo de Especialidades Químicas, S.A. de C.V. (Nuevo León, Mexico). Trypsin–EDTA 0.25% and fetal bovine serum (FBS) were purchased from HyClone, Thermo Scientific (Logan, UT, USA). Agarose and GelRed™ nucleic acid stain were acquired from Biotium (Fremont, CA, USA). Restriction enzymes XbaI and BlpI were obtained from New England Biolabs (Ipswich, MA, USA). Synthetic T7 primers were purchased from Integrated DNA Technologies (Coralville, IA, USA).

LB broth and LB agar were obtained from Difco, BD (Sparks, MD, USA).

Immobilon PVDF (polyvinylidene difluoride) membranes and 0.22 µm polyethersulfone (PES) syringe filters were purchased from Millipore (Burlington, MA, USA). HRP-conjugated anti-6 × His monoclonal antibody was obtained from Santa Cruz Biotechnology (Dallas, TX, USA). *Centella asiatica* extract was purchased from Selleck Chemicals (Houston, TX, USA).

### Biological material

Human breast adenocarcinoma (MCF-7), human colorectal adenocarcinoma (Caco-2), and human primary dermal fibroblasts (HDFa) cells were purchased from the American Type Culture Collection (ATCC®, Manassas, VA, USA). *Escherichia coli* BL21(DE3) cells, used for transformation, were obtained from Thermo Fisher Scientific *(Waltham, MA, USA).*

### Plasmid design and construction

**Fig. 1:**
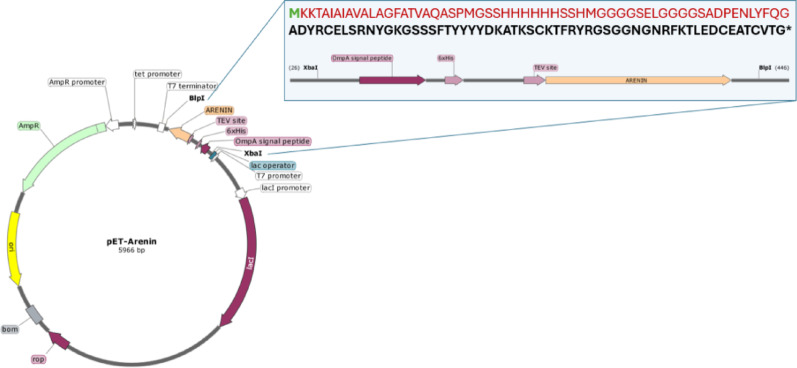
pET-Arenin plasmid. The construct includes the *arenin* gene under a T7 promoter, fused to an OmpA signal peptide, a 6xHis tag (Hexahistidine tag), and a TEV protease site, cloned between the XbaI and BlpI restriction sites. The inset shows the amino acid sequence of the expressed fusion protein (Green: start codon; Red: upstream regulatory and fusion tags; Black: arenin)

The sequence encoding arenin (UniProt Entry: A0A455MWG9), a peptide derived from *Dryophytes arenicolor*, was synthesized using the GeneSmart platform (GenScript, Piscataway, NJ, USA). To facilitate downstream purification, a 6 × His tag was added at the N-terminus, followed by a TEV (Tobacco etch virus) protease recognition site to enable potential tag removal. Additionally, the *ompA* signal sequence was included upstream of the arenin coding region to direct translocation to the periplasmic space. The codon-optimized sequence for *Escherichia coli* expression was cloned into the pET28a( +) vector between the *XbaI* and *BlpI* restriction sites, under the control of an IPTG-inducible T7 promoter (Fig. 1). Correct insertion and fragment size were verified by restriction digestion and agarose gel electrophoresis (1.5% agarose stained with GelRed; Biotium).

### Plasmid propagation and validation

Plasmids were propagated in *E. coli* DH5α cells made competent using the RbCl₂ method. A single colony was cultured in LB medium supplemented with 50 µg mL^−1^ kanamycin at 37 °C and 250 rpm. This pre-culture was scaled up to 100 mL under the same conditions until reaching OD_600_ ≈ 0.4. Cells were chilled on ice, harvested, and resuspended in transformation buffers I and II. Aliquots (50 µL) were stored at − 80 °C. For transformation, 25 ng of plasmid DNA were mixed with competent cells, heat-shocked, recovered in SOC medium, and plated on LB-kanamycin agar. Glycerol stocks (15%) were prepared from overnight cultures for future use. Expression vector verification was performed by miniprep purification (Wizard Plus SV Miniprep Kit, *Promega, Madison, WI, USA*), followed by linearization using XbaI and BlpI (New England Biolabs). The digested DNA was purified from agarose gel with the Wizard SV Gel and PCR Clean-up Kit (*Promega, Madison, WI, USA*). Colony PCR was performed using T7-specific primers (Forward: 5′-CGAAATTAATACGACTCACTATAGGGG-3′, Reverse:5′ GGGTTATGCTAGTTATTGCTCAGC-3′). Amplifications were performed in 10 µL reactions using GoTaq Green MM (Promega) with the following cycling conditions: 95 °C for 5 min; 30 cycles of 95 °C for 30 s, 50.5 °C for 30 s, and 72 °C for 3 min, with a final extension at 72 °C for 5 min. Products were resolved on 1% agarose gels. Additionally, plasmid sequence integrity was confirmed by Sanger sequencing (GenScript, USA) using internal primers flanking the arenin gene. The resulting electrophegrams were analyzed using SnapGene version 8.1.1 and aligned with the designed construct sequence. No mutations, insertions, or deletions were detected in the coding region or regulatory elements.

### Recombinant expression of arenin and clone screening

Plasmids confirmed by Sanger sequencing were transformed into *E. coli* BL21 Star™ (DE3) via CaCl₂ heat-shock. To rigorously compare induction temperatures, we used three independent biological replicates per condition. For each replicate, a single colony was inoculated into 5 mL LB + 100 µg mL^−1^ kanamycin and grown overnight at 37 °C/250 rpm. These pre-cultures were each used to inoculate 25 mL fresh LB + kanamycin in 250 mL baffled flasks, which were then split into two groups: one incubated at 30 °C, the other at 37 °C (250 rpm). Growth was monitored by measuring OD_600_ every 30 min, and when cultures reached OD_600_ ≈ 0.6, expression was induced with 0.5 mM IPTG. Post-induction samples (1 mL) were taken at 0, 4, and 8 h to track expression kinetics via SDS-PAGE. After 8 h, cells were harvested (5000 × *g*, 10 min, 4 °C), pellets flash-frozen in liquid nitrogen, and stored at − 80 °C.

For clone and solubility screening, pellets were thawed and lysed in 50 mM Tris–HCl, 300 mM NaCl, 8 M urea, pH 8.0. The insoluble fraction was fully solubilized by gentle agitation for 1 h at room temperature. Five microliters of each lysate (in duplicate) were spotted onto methanol-activated PVDF membranes (PBS-T 0.1%), then blocked with 5% skim milk in PBS-T for 1 h. Membranes were incubated with His-tag HRP-conjugated antibody (1:1000; Santa Cruz) for 1 h, washed three times in PBS-T, and developed using Clarity™ ECL substrate (Bio-Rad). Chemiluminescence was captured on an iBright™ system (Applied Biosystems), and spot intensities were quantified by densitometry to determine the proportion of soluble versus insoluble arenin under each temperature regime. This design allowed precise identification of 30 °C/8 h as the condition yielding maximal soluble protein.

### Protein extraction and IMAC purification

Cell pellets were resuspended in lysis buffer (20 mM sodium phosphate, 500 mM NaCl, 20 mM imidazole, pH 7.4) and lysed on ice by sonication (QSONICA Q125; 5 × 15 s pulses, 60% amplitude, 30 s rests). After centrifugation (12,000 × g, 20 min, 4 °C), the supernatant (soluble fraction) was filtered (0.22 µm) and loaded onto a HisTrap HP 1 mL column (Cytiva) equilibrated with 15 column volumes (CV) of Equilibration Buffer 1 (A1) 50 mM NaH₂PO₄, 300 mM NaCl, 20 mM imidazole, pH 7.4 at 1 mL min^−1^. The column was washed with 5 CV of A1 at 1 mL min^−1^, followed by 10 CV of A1 at 1 mL min^−1^, and arenin was eluted with 5 CV of Elution Buffer (B1) (50 mM NaH₂PO₄, 300 mM NaCl, 300 mM imidazole, pH 7.4. 1 mL min^−1^) at 1 mL min^−1^.

The insoluble pellet was solubilized in Equilibration Buffer 2 (B2) (50 mM NaH₂PO₄, 300 mM NaCl, 20 mM imidazole, 8 M urea, pH 7.4) for 2 h at room temperature, clarified by 0.22 µm filtration, and loaded onto a HisTrap HP 1 mL column pre-equilibrated with 15 CV of B2 at 1 mL min^−1^. A linear gradient from B2 to A1 was applied over 15 CV at 1 mL min^−1^. The column was then washed with 8 CV of A1 at 1 mL min^−1^, followed by 10 CV of A1 containing 7.1% B1 at 1 mL min^−1^, and arenin was eluted with 10 CV of B1 at 1 mL min^−1^.

Eluted fractions from the soluble and insoluble purifications were processed separately. Each fraction containing arenin was subjected to TEV protease cleavage to remove the N-terminal His-tag. Cleavage was performed in 1 × TEV Protease Reaction Buffer (50 mM Tris–HCl, 0.5 mM EDTA, 1 mM DTT, pH 7.5 at 25 °C) using 1 unit of TEV protease per 2 µg of substrate, in a total reaction volume adjusted accordingly. Reactions were incubated at 30 °C for 1 h to achieve ≥ 95% cleavage. Following digestion, each reaction mixture was loaded onto a second IMAC purification using a HisTrap HP 1 mL column to remove the His-tag, uncleaved protein, and His-tagged TEV protease. The flow-through containing tag-free arenin was collected, desalted using PD-10 Sephadex G-25 columns (Cytiva), concentrated, lyophilized, and quantified using the bicinchoninic acid (BCA) assay.

### SDS-PAGE analysis

Protein expression and purity were assessed by SDS-PAGE. Samples (15 µL) of crude lysate, soluble/insoluble fractions, and purified protein were mixed with 3 µL of Laemmli loading buffer, boiled for 5 min, and loaded onto 12% Tris–Glycine gels (Mini-PROTEAN Tetra Cell, Bio-Rad). A Precision Plus Protein™ Dual Color ladder (Bio-Rad) was included to calibrate molecular weights. Gels were run at 60 V (stacking) then 100 V (resolving), stained with colloidal Coomassie Blue, and imaged on an iBright FL1500 system (Thermo Fisher). Band identity was further validated by correlation with the expected size. The purity was calculated by optical densitometry: band intensities were quantified using iBright Analysis Software (Thermo Fisher) and expressed as the percentage of total lane signal contributed by the arenin band. Finally, protein concentration was determined using a bicinchoninic acid (BCA) assay, completing a comprehensive analysis of arenin yield and purity.

### Serine protease BAEE activity assay

To confirm that heterologously expressed arenin was correctly folded and exerted its functional protease-inhibitory capacity, an intrinsic characteristic of all Kunitz-type peptides, a serine protease inhibition assay based on Song et al. (2018) with slight modifications was conducted. Arenin (0 to 11.57 µg mL-1) was incubated with 1 µg of recombinant porcine trypsin (RTYP-RO, Sigma Aldrich, San Luis, USA) in 20 µL of Tris–HCl 10 mM, pH 7.4, at 37 ºC for 30 min. Then, 100 µL of 0.5 mM BAEE (Nα-Benzoyl-L-arginine ethyl ester hydrochloride, Sigma-Aldrich, San Luis, USA) substrate was added to initiate the hydrolysis reaction. Serine protease activity was monitored for 4 min at 253 nm with a spectrophotometer (Synergy HT, Bio-Tek, Winooski, USA at 37 ºC. Enzymatic units are defined as an increase of 0.001 at 253 nm absorbance in 1 min. All reactions were done in triplicate. Arenin concentrations (0–11.67 µg mL^−1^) were selected based on preliminary inhibition trials (data not shown) to span the transition from no inhibition to complete inhibition, consistent with concentration ranges commonly used for peptide-based serine protease inhibitors. IC_50_ was calculated by fitting concentration–response data to a four-parameter logistic (4PL). The activity percentage was calculated as the proportion of the arenin-treated samples to the untreated serine protease reaction.

### In vitro cytotoxicity and antitumoral activity of recombinant arenin

A stock solution was prepared by dissolving the lyophilized arenin in sterile water at a concentration of 1 mg mL^−1^, using moderate stirring to ensure complete dissolution. The solution was then sterilized by passing it through a 0.22 µm filter. After filtration, the peptide concentration was verified using a BCA protein assay kit. Human dermal fibroblasts (HDFa), breast adenocarcinoma cells (MCF-7), and colorectal adenocarcinoma cells (Caco-2) were seeded at a density of 5 × 10^4^ cells/mL in a 96-well plate and incubated for 24 h at 37 °C in a 5% humidified CO_2_ atmosphere. After, cells were treated with different concentrations of arenin (31.25–1000 µg mL^−1^) in Dulbecco's Modified Eagle's Medium (DMEM) F12 supplemented with 5% FBS and 1% penicillin/streptomycin and incubated for 48 h. Cell viability assay was performed using the MTS [3-(4,5-dimethylthiazol-2-yl)-5-(3-carboxymethoxyphenyl)-2-(4-sulfophenyl)-2H-tetrazolium)]-based CellTiter 96 Aqueous One Solution Cell Proliferation assay. MTS reagent was added to each well, and the plate was incubated for 45 min at 37 °C. Following this incubation, absorbance was recorded at 490 nm using a 96-well microplate reader (Synergy HT, Bio-Tek, Winooski, VT, USA). The concentration range (31.25–1000 µg mL^−1^) followed a standard two-fold serial dilution approach widely applied in MTS-based cytotoxicity assays for peptides and extracts. Cell viability was expressed as a percentage relative to the untreated control cells. Before the FBS (−) deprivation and high-glucose (HG) experiments, a preliminary cell-viability screen was performed, and only those arenin concentrations that maintained ≥ 80% viability were subsequently selected for the assays.

### In vitro wound-healing activity of recombinant arenin

The scratching assay was performed using human dermal fibroblasts (HDFa) cells seeded at 150,000 cells/well in 24-well plates and incubated overnight in culture medium. Artificial wounds (0.5 cm wide) were created using sterile 200 µL pipette tips, followed by a gentle wash with PBS. Subsequently, wounds were treated with arenin diluted in Dulbecco’s Modified Eagle Medium (DMEM) supplemented with 5% fetal bovine serum (FBS) (31.25 µg mL^−1^ to 1000 µg mL^−1^) in triplicate, refreshing treatment every 24 h. Images were captured at 0, 24, 48, and 72 h using an Olympus CK2 microscope. *Centella asiatica* commercial extract (10 µg mL^−1^) was used as a positive control. Wound area measurements were performed using the Fiji Wound Healing Size Tool. The wound closure percentage (WC%) was calculated according to Eq. 1, where T₀ represents the initial time point (0 h), and Tₙ corresponds to the evaluated time point (e.g., 24, 48, or 72 h). WC% indicates the percentage reduction of the wound area relative to the original wound size.

1 $$ WC\% = \frac{Wound\;area\;at\;To - Wound\;area\;at\;Tn}{{Wound\;area\;at\;To}} \times 100 $$

### Statistical analysis

All measurements were performed at least in triplicate. Data analysis was conducted using Minitab software, and a *p*-value of < 0.05 was considered to indicate statistical significance. Results are presented as the mean ± standard deviation (S.D.). Differences between the groups were determined by one-way analysis of variance (ANOVA) followed by a Tukey post hoc test for multiple comparisons.

## Results and discussion

### Plasmid verification and clone screening

**Fig. 2:**
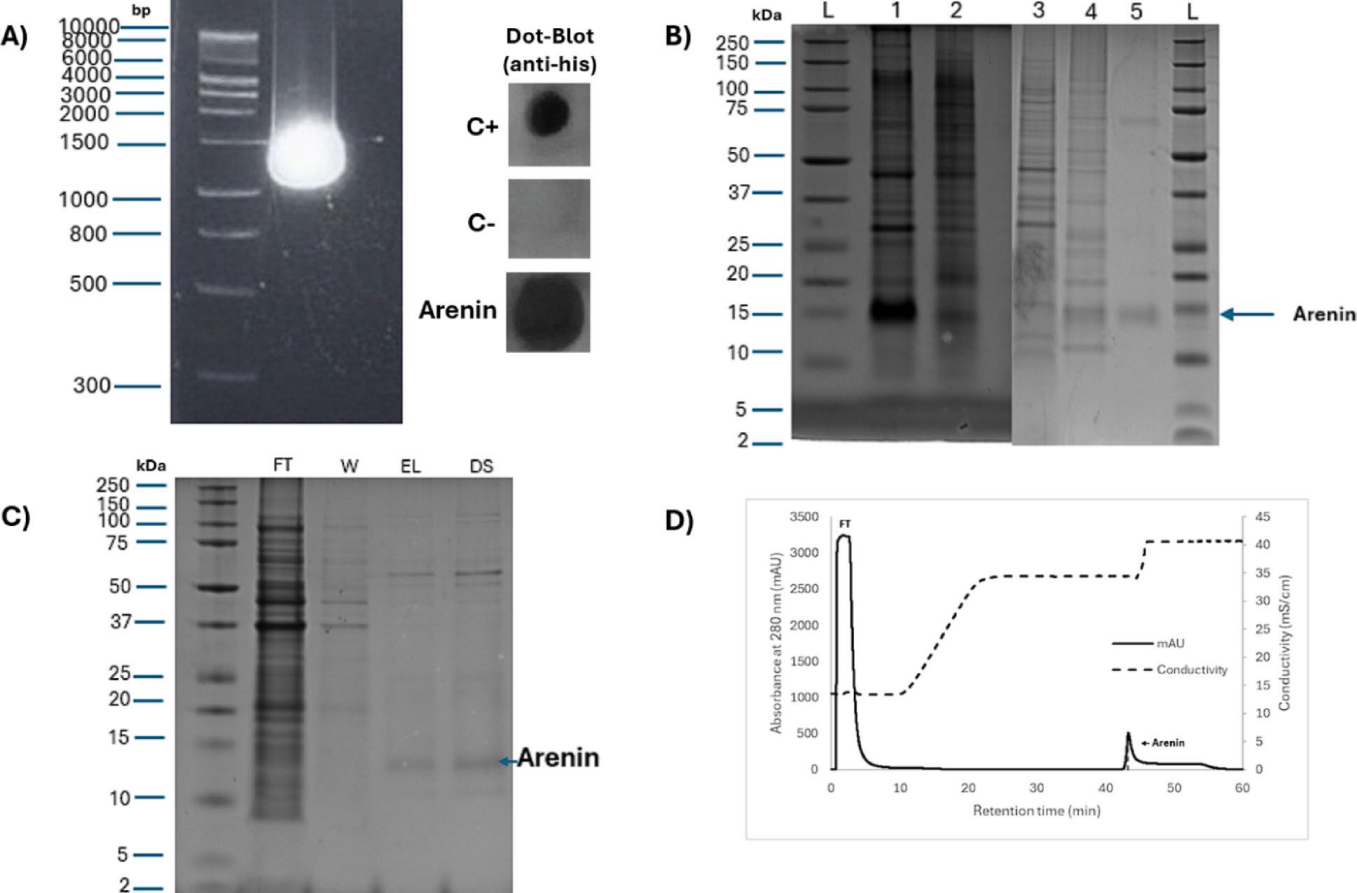
Heterologous expression and purification of recombinant arenin. **A** Verification of arenin gene insertion (1461 bp) by PCR and confirmation of expression in IPTG-induced clones by Dot-Blot analysis. **B** SDS-PAGE analysis demonstrating the presence of arenin (~ 15 kDa) exclusively in induced bacterial cultures. Lane L, molecular weight marker; lane 1, IPTG-induced bacterial culture; lane 2, non-induced culture; lanes 3 and 4, soluble and insoluble fractions, respectively; lane 5, final purified arenin after IMAC. **C** SDS-PAGE illustrating the stepwise enrichment of arenin through a HisTrap HP 1 mL column, notably in the elution (EL) and subsequent desalting (DS) fractions. **D** Representative IMAC chromatographic profile under optimized conditions, highlighting arenin elution as a sharp, symmetrical peak. FT indicates the flow-through fraction, W represents the wash step, EL corresponds to the elution fraction, and DS refers to the desalting fraction

Plasmid propagation was performed in RbCl_2_-competent *E. coli* DH5α cells, yielding 80–120 µg of purified plasmid per 100 mL of LB + 100 µg mL^−1^ kanamycin, in line with lab-scale culture benchmarks. The presence and integrity of the arenin insert were confirmed by endpoint PCR amplification using specific T7 primers, resulting in the expected 1461-bp amplicon (Fig. 2A). The PCR product was visualized by agarose gel electrophoresis and matched the predicted size of the arenin coding region, indicating successful cloning and plasmid propagation for downstream expression. To further confirm construct integrity, the plasmid was sequenced across the arenin insert and its regulatory regions.

The sequence matched the expected design without any mutations, confirming the correct assembly of the recombinant pET-Arenin plasmid (Fig. 1). Sanger sequencing across the insert and regulatory elements revealed 100% identity to the designed construct, verifying the correct in-frame fusion of the OmpA signal peptide, 6 × His tag, TEV site, and arenin open reading frame. Comparable sequence-based validation is routinely employed to pre-empt expression failures caused by spontaneous mutations in T7-based plasmids (Hernández-Pérez et al. 2018; Rigi et al. 2021).

Following plasmid validation, the selection of productive arenin-expressing clones was performed via Dot blot analysis targeting the insoluble protein fraction. After induction, cell pellets were lysed, and the insoluble material was solubilized using 8 M urea. PVDF membranes were spotted with these preparations and probed with His-tag antibodies conjugated to HRP. Dot blotting offers a < 1 h, high-throughput alternative to SDS-PAGE for detecting affinity-tagged proteins (Ibraheem et al. 2011; Mishra 2022), and has been recommended for inclusion-body screens where rapid clone triage is critical. Among the clones screened, several exhibited strong chemiluminescent signals compared to non-induced controls (Fig. 2A, lateral right panel), confirming successful intracellular expression of His-tagged arenin in inclusion bodies (Singh et al. 2015). The clone exhibiting the strongest signal was selected for further recombinant arenin expression cultures.

Arenin is composed of 58 amino acids and has a predicted molecular weight of approximately 6.8 kDa based on its primary sequence. However, SDS-PAGE analysis consistently revealed a single dominant band migrating at ~ 15 kDa (Fig. 2B and C), suggesting a clear discrepancy between its theoretical and apparent molecular mass. This migration shift, commonly referred to as a “gel shift,” is frequently observed in compact, disulfide-rich proteins and is often attributed to incomplete SDS binding, altered charge-to-mass ratios, or residual oligomerization during electrophoresis (Rath et al. 2009). Additionally, histidine-rich peptides may exhibit similar anomalous migration due to interactions with Ni^2^⁺ ions that persist through SDS-PAGE (Shelake et al. 2017). In the case of arenin, this atypical migration behavior is consistent with its tightly folded Kunitz-type structure and the presence of an N-terminal His-tag. Importantly, correct folding was further supported by functional serine-protease inhibition (BAEE/trypsin) (Fig. 4), showing a clear dose-dependent response and complete inhibition at the highest concentration tested an activity that requires the native disulfide-bonded Kunitz fold. These results provide functional evidence that recombinant arenin retains its expected tertiary structure despite its anomalous electrophoretic migration. Notably, the ~ 15 kDa band was reproducibly detected across both soluble and insoluble fractions and corresponded precisely with the expected expression and purification profiles, confirming that this band indeed represents the expressed recombinant arenin. While additional structural analyses such as circular dichroism or disulfide-mapping mass spectrometry could further support functional characterization, the techniques employed in this study provide sufficient validation of the observed biological activity.

### Expression of recombinant arenin

The expression of recombinant arenin was assessed by comparing two induction temperatures, 30 °C and 37 °C, to gain insights into its solubility profile and overall expression efficiency. The observed production levels are consistent with laboratory-scale benchmarks reported for other cysteine-rich peptides (0.1–1.0 mg mL^−1^) (Pouresmaeil and Azizi-Dargahlou 2023). To better understand the impact of induction temperature on arenin solubility and intracellular distribution, total protein and arenin content were quantified across three fractions: cell lysate, soluble fraction, and insoluble fraction (Table 1). Induction at 37 °C maximized total protein synthesis, yielding 400.0 ± 8.52 mg L^−1^, with arenin accounting for 126.4 ± 3.9 mg L^−1^ (32.3% of total protein). At this temperature, only 44.0 ± 3.4 mg L^−1^ (22.0%) was soluble, whereas 85.2 ± 6.4 mg L^−1^ (42.5%) accumulated in inclusion bodies, reflecting common challenges related to folding disulfide-rich proteins in the reducing cytoplasm of bacterial hosts (Bhatwa et al. 2021).

**Table 1:** Effect of induction temperature on the expression and solubility fraction distribution of arenin in *E. coli*

30 °C				
Step	Total protein (mg L^−1^)	Arenin (mg L^−1^)	Arenin purity (%)	Arenin yield (%)
Cell lysis	249.6 ± 6.6	85.9 ± 2.5	34.5	100.0
Soluble fraction	143.6 ± 30.4	59.2 ± 4.8	41.2	69.1
Insoluble fraction	106.0 ± 16.0	27.1 ± 4.4	25.6	31.5

37 °C				
Step	Total protein (mg L^−1^)	Arenin (mg L^−1^)	Arenin purity (%)	Arenin yield (%)
Cell lysis	400.0 ± 8.5	126.4 ± 3.9	32.3	100.0
Soluble fraction	200.0 ± 30.0	44.0 ± 3.4	22.0	34.8
Insoluble fraction	200.0 ± 32.0	85.2 ± 6.4	42.5	67.7

Results correspond to cultures grown in 250 mL of LB medium. Data represent mean ± SD (n = 3) for total protein content, arenin amount, relative purity (%), and yield (%) in each fraction obtained after cell lysis

In contrast, induction at 30 °C resulted in a lower total protein yield of 249.6 ± 6.69 mg L^−1^, but a markedly improved solubility profile: arenin was recovered at 59.2 ± 4.8 mg L^−1^ in the soluble fraction (41.2% purity) versus 27.1 ± 4.48 mg L^−1^ in inclusion bodies (25.6% purity). This enhanced solubility at lower temperature aligns with reports that slower translation rates at ≤ 30 °C facilitate co-translational folding and reduce aggregation (Rosano and Ceccarelli 2014; Sørensen and Mortensen 2005). SDS-PAGE analysis (37 °C) (Fig. 2B) of induced cultures (lane 1) revealed a prominent protein band at approximately 15 kDa, indicative of recombinant arenin expression. This band was absent in non-induced control samples (lane 2), confirming IPTG-dependent expression. Arenin predominantly accumulated in inclusion bodies (lane 4) rather than the soluble fraction (lane 3), consistent with its accumulation in inclusion bodies. The purified protein (lane 5) showed a single band, supporting the specificity of Ni–NTA affinity purification. Densitometric analysis estimated arenin to constitute approximately 32% of the total protein (lane 1), while reaching over 90% purity after purification (lane 5). This expression profile is comparable to that reported for other recombinant Kunitz-type inhibitors, such as SKTI and anticoagulant peptides (Francis and Page 2010; Sun et al. 2024).

### Purification of recombinant arenin

Recombinant arenin was purified from both soluble and insoluble fractions of *E. coli* cultures induced at 30 °C and 37 °C. Post-lysis and clarification, soluble fractions were subjected to Ni–NTA affinity chromatography under native conditions, while insoluble fractions were denatured, refolded, and subsequently purified under denaturing/renaturing protocols. The chromatographic workflow followed a classical bind–wash–elute scheme using IMAC with imidazole step gradients ranging from 10 to 300 mM, and phosphate buffer supplemented with 300 mM NaCl to enhance selectivity and reduce nonspecific interactions (Fig. 2C) (Spriestersbach et al. 2015). The IMAC chromatographic profile showed a clean separation of arenin as a sharp peak (Fig. 2D), confirming optimal purification conditions and efficient recovery of histidine-tagged protein.

Quantitative purification results for each condition are summarized in Table 2 as a mass balance, including protein yields, purity levels, recovery percentages, and purification factors (PF), and track each of the four fractions obtained in the expression experiments (Table 1). At 37 °C, purification from the soluble fraction yielded 5.1 ± 0.45 mg of arenin at 92.7% purity (87.9% stage recovery, PF = 4.2). In contrast, arenin recovered from the refolded insoluble fraction reached 12.4 ± 1.02 mg at 93.9% purity, corresponding to a 39.7% overall recovery and a PF of 2.2. These values are comparable to those reported for refolded plant-derived Kunitz inhibitors such as AKPI 1 and AKPI 2 from *Apios americana* (Bonturi et al. 2022), highlighting the feasibility of using inclusion bodies as a viable protein source.

**Table 2:** Mass balance of recombinant arenin purification from soluble and insoluble fractions obtained at 30 °C and 37 °C

Step	Soluble route (30 °C)					
	Total protein (mg)	Arenin (mg)	Arenin purity (%)	Step Yield (%)	Arenin yield (%)	PF
Soluble fraction	35.9 ± 7.5	14.8 ± 1.1	41.2	–	69.1	1.0
Affinity chromatography	12.1 ± 0.3	9.2 ± 0.7	76.0	62.1	42.9	1.8
TEV cleavage	10.0 ± 0.5	8.8 ± 0.7	88.0	95.6	41.1	2.1
2nd Ni–NTA	9.6 ± 0.6	8.5 ± 0.6	88.5	96.5	39.7	2.1
Desalting (PD-10)	8.5 ± 0.4	7.8 ± 0.6	91.7	91.7	36.4	2.2

Step	Insoluble route (30 °C)					
	Total protein (mg)	Arenin (mg)	Arenin purity (%)	Step Yield (%)	Arenin yield (%)	PF
Insoluble fraction	26.5 ± 5.0	6.7 ± 0.7	25.6	–	31.5	1.0
Affinity chromatography	5.3 ± 1.0	3.8 ± 0.5	71.7	56.0	17.7	2.8
TEV cleavage	4.4 ± 1.0	3.7 ± 0.5	84.0	97.4	17.2	3.3
2nd Ni–NTA	4.2 ± 0.9	3.45 ± 0.5	82.1	93.2	16.1	3.3
Desalting (PD-10)	3.8 ± 0.8	3.2 ± 0.4	84.2	92.7	15.0	3.3

Step	Soluble route (37 °C)					
	Total protein (mg)	Arenin (mg)	Arenin purity (%)	Step Yield (%)	Arenin yield (%)	PF
Soluble fraction	50.0 ± 7.7	11.0 ± 0.8	22.0	–	34.8	1.0
Affinity chromatography	9.5 ± 0.6	6.5 ± 0.5	68.4	59.0	20.6	3.1
TEV cleavage	6.62 ± 0.6	5.9 ± 0.5	89.0	90.8	18.7	4.0
2nd Ni–NTA	6.17 ± 0.6	5.8 ± 0.5	94.0	98.3	18.4	4.2
Desalting (PD-10)	5.5 ± 0.5	5.1 ± 0.4	92.7	87.9	16.1	4.2

Step	Insoluble Route (37 °C)					
	Total Protein (mg)	Arenin (mg)	Arenin purity (%)	Step yield (%)	Arenin yield (%)	PF
Insoluble fraction	50.0 ± 8.0	21.3 ± 1.6	42.5	–	67.7	1.0
Affinity chromatography	18.7 ± 2.0	15.0 ± 1.2	80.0	70.6	47.5	1.8
TEV cleavage	14.9 ± 1.9	13.5 ± 1.1	90.2	90.0	42.7	2.1
2nd Ni–NTA	14.6 ± 1.8	13.2 ± 1.1	90.0	97.8	41.8	2.1
Desalting (PD-10)	13.2 ± 1.7	12.4 ± 1.0	93.9	93.9	39.7	2.2

Balance corresponds to 250 mL cultures. Data represent mean ± SD (*n* = 3) for total protein (mg), arenin amount (mg), arenin purity (%), step yield (% relative to the previous stage), cumulative yield (% relative to cell lysate), and purification factor (PF), calculated as the ratio of purity at each stage to the initial purity in the corresponding fraction

Interestingly, at 30 °C, arenin purification from the soluble fraction yielded a higher recovery (7.8 ± 0.63 mg; 91.7% purity, 36.4% yield, PF = 2.2), while the insoluble route produced 3.2 ± 0.45 mg (84.2% purity, 15.0% yield, PF = 3.3). These findings indicate that temperature modulation alone can significantly impact protein folding and purification outcomes, with lower induction temperatures promoting soluble expression and reducing the dependency on complex folding protocols. This temperature–solubility trade-off is consistent with previous studies on spider venom-derived Kunitz toxins, which exhibit enhanced functional activity and native folding when expressed at 25–30 °C compared to higher temperatures (Alvarado et al. 2021).

**Fig. 3:**
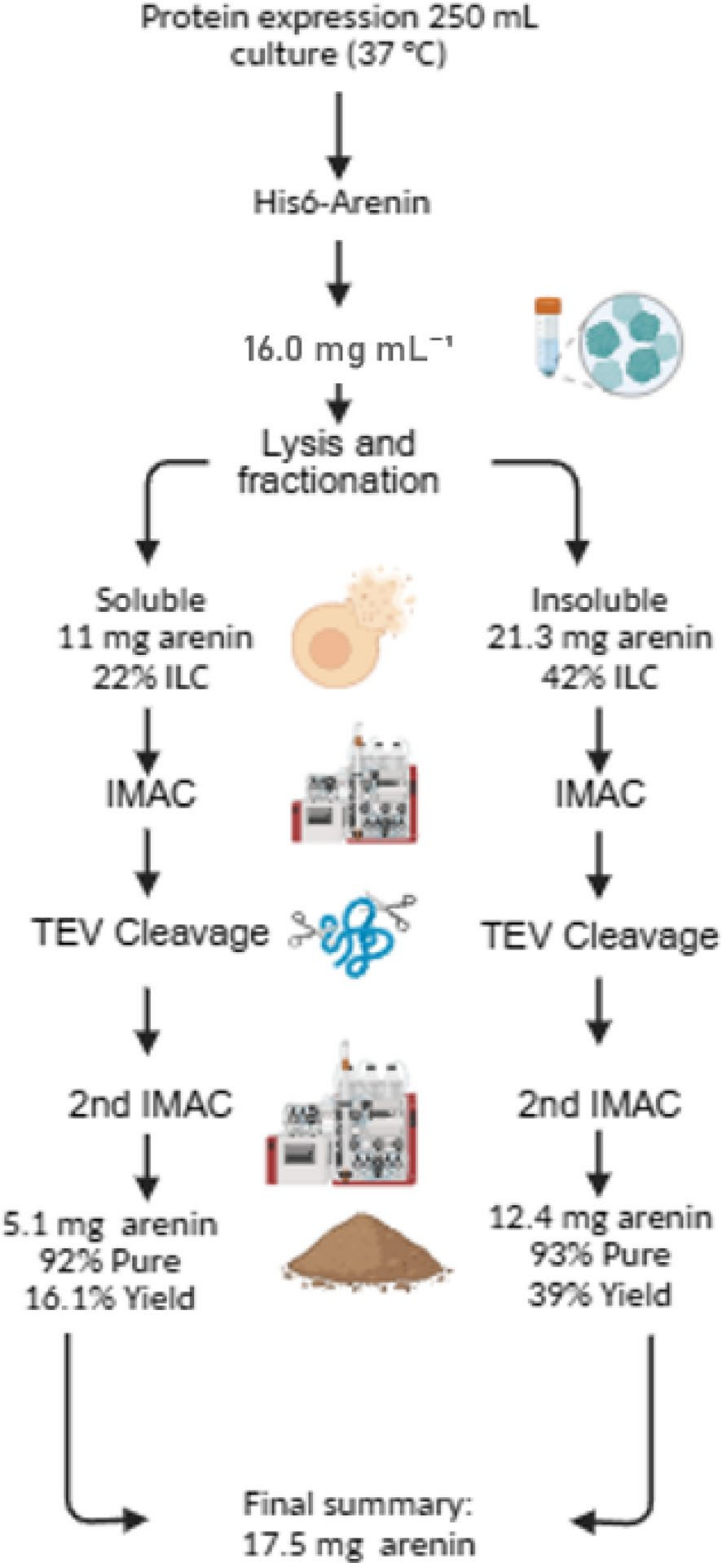
Flow diagram of the selected optimal process among evaluated conditions for recombinant arenin production induced at 37 °C. The workflow includes cell lysis, protein fractionation, tandem immobilized metal affinity chromatography (IMAC), and proteolytic cleavage using tobacco etch virus (TEV) protease. ILC, intracellular content. All concentrations are reported in mg L^−1^ relative to the original culture volume (250 mL). Illustration created with BioRender.com

Despite the higher soluble recovery observed at 30 °C, the 37 °C induction condition combined with inclusion body capture yielded greater total arenin per 250 mL culture, while shortening cultivation time and reducing temperature-control complexity. This configuration enabled a robust, efficient, and operationally simple IMAC–TEV–IMAC workflow. Considering the trade-off between solubility and volumetric productivity, the 37 °C protocol was selected as the preferred route for Fig. 3, as it maximizes total arenin output while preserving downstream purity and reproducibility. The IMAC–TEV–IMAC purification pipeline employs standard, linearly scalable chromatographic operations, with preserved column volumes (CVs), residence times, and buffer chemistries across scales, enabling direct transfer to liter-scale or pilot-scale systems. While downstream operations generally scale with minimal loss of performance, upstream expression yields can be affected by reactor size due to differences in oxygen transfer, mixing, and metabolic gradients. Although the present work was conducted at laboratory scale, the selected host (*E. coli*), expression system (T7), and inclusion body strategy are commonly used in industrial settings and are well suited for high-density, fed-batch fermentation. Therefore, the process pipeline, from heterologous expression to purification has scaling-up feasibility. Although scaling-up the process may have some impact over overall yields, the control of key process parameters may allow to achieve results as those obtained at lab scale.

### Recombinant arenin inhibition over serine protease activity

Kunitz-type peptides are a well-characterized family of serine protease inhibitors widely distributed across animals, plants, and microorganisms. These peptides typically exert their function by forming non-covalent, high-affinity complexes with target proteases, thereby blocking substrate access to the active site in a manner that closely resembles a canonical enzyme–substrate interaction (Ranasinghe and McManus 2013). Due to this mechanism, Kunitz inhibitors play essential roles in physiological processes such as inflammation, coagulation, and tissue remodeling. Arenin, previously isolated from the skin secretion of *Dryophytes arenicolor*, has been identified as a Kunitz-like peptide with conserved cystine-rich motifs characteristic of this inhibitor family (Hernández-Pérez et al. 2018). Given its structural features, we sought to evaluate whether the expressed recombinant arenin exhibits inhibitory activity against serine proteases, using porcine trypsin and the synthetic substrate BAEE (Nα-Benzoyl-L-arginine ethyl ester) as a model system.

**Fig. 4:**
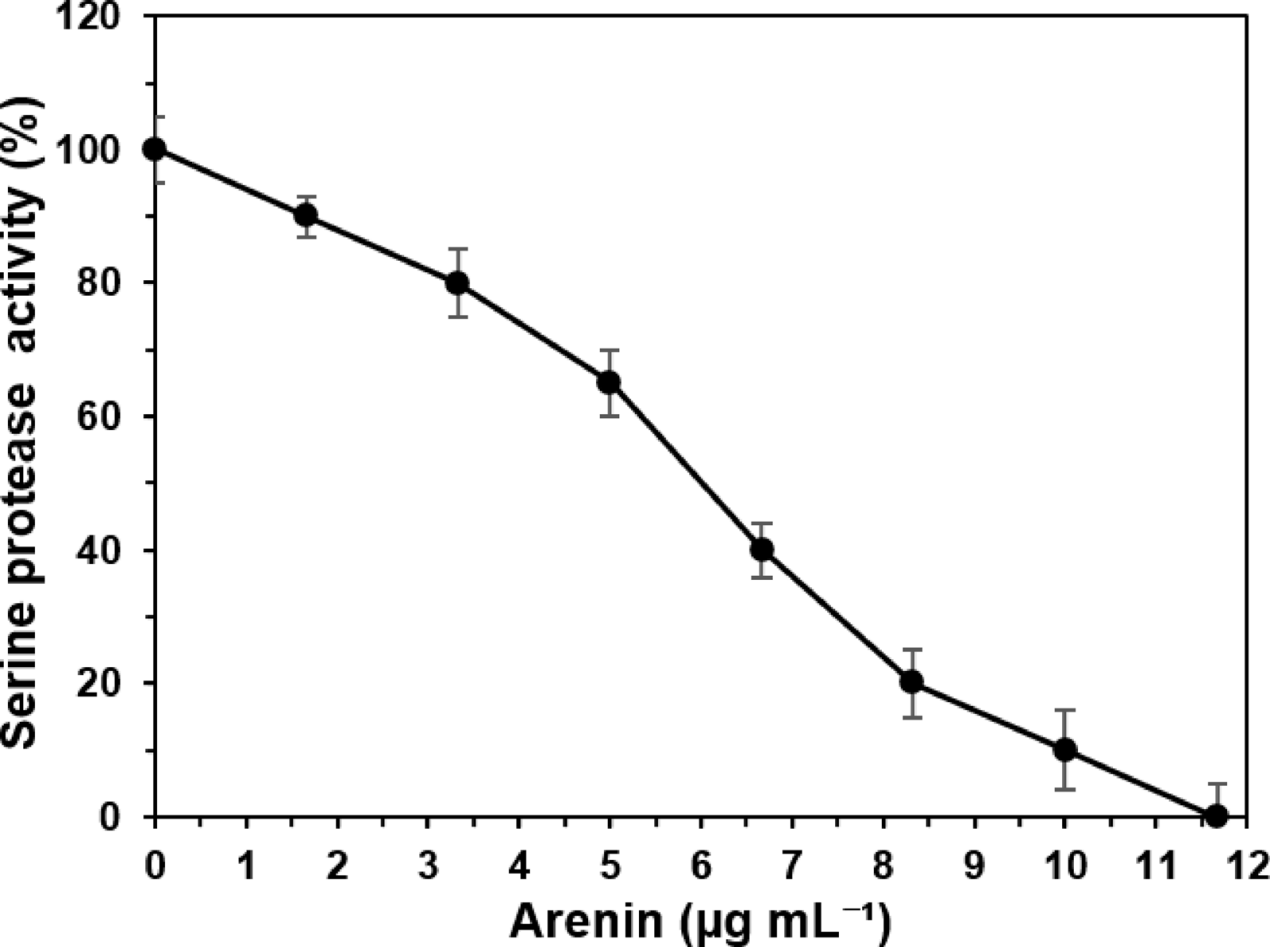
Effect of recombinant arenin on recombinant porcine trypsin activity using BAEE (Nα-Benzoyl-L-arginine ethyl ester) as substrate. A volume of 20 µL of arenin (0.00, 1.67, 3.33, 5.00, 6.67, 8.33, 10.00, 11.67 µg mL^−1^) and trypsin (1 µg) were added to 100 µL of BAEE 0.5 mM Tris–HCl 10 mM pH 7.4 and let to react at 37 ºC. BAEE hydrolysis was monitored at 253 nm for 4 min at 37 ºC. Each point represents the mean ± standard deviation of triplicates. IC_50_ = 6.04 µg mL^−1^

Through this experiment, it was observed that the expressed recombinant arenin inhibits serine protease activity in a dose-dependent manner, with complete inhibition achieved at 11.67 µg mL^−1^ (equivalent to 1.4 µg; Fig. 4). Nonlinear regression of the concentration–response curve using a four-parameter logistic (4PL) model yielded an IC_50_ of 6.04 µg mL^−1^ for recombinant arenin. This value places arenin within the expected potency range of Kunitz-type serine protease inhibitors targeting trypsin. For instance, a 20 kDa Kunitz inhibitor purified from *Gymnocladus chinensis* seeds exhibited an IC_50_ of approximately 0.4 µM (equivalent to ~ 8 µg mL^−1^), demonstrating comparable inhibitory capacity under enzymatic assay conditions (Zhu et al. 2011). Likewise, rusvikunin, a venom-derived Kunitz inhibitor from *Daboia russelii*, showed high-affinity inhibition of trypsin with an IC_50_ of ~ 50 nM (~ 0.35 µg mL^−1^), illustrating the potent activity achievable within this structural family (Mukherjee et al. 2014).These results demonstrate that the recombinant arenin behaves as a potent serine protease inhibitor within a narrow concentration range and may possess similar inhibitory strength as other Kunitz-type peptides. Comparable inhibition profiles have been reported for Kunitz-type peptides from amphibians and arthropods, where complete inhibition occurs in the sub- to low-micromolar range. For instance, recombinant *Araneus ventricosus* Kunitz peptide showed maximal trypsin inhibition at 0.6 to 2 µg (Wan et al. 2013).

In contrast, other Kunitz peptides, such as bungarokunin, isolated from *Bungarus fasciatus* venom, demonstrated a weaker effect, requiring 27 µg mL^−1^ to reach 10% residual trypsin activity (Lu et al. 2008). Compared to these, arenin demonstrates a potent inhibition profile at equivalent or even lower concentrations.

The potent inhibitory capacity of arenin might be attributed to its structural homology to other Kunitz inhibitors, which are known to bind the active site of serine proteases tightly. However, it is also essential to consider that homodimerization, as previously reported for arenin, could influence its inhibitory potential depending on concentration and folding conditions (Hernández-Pérez et al. 2018).

The sigmoidal curve observed in the dose–response profile suggests a classical inhibitory mechanism, possibly reflecting a single binding event per enzyme molecule, characteristic of tight-binding competitive inhibitors. This pattern has also been observed for other peptide inhibitors tested against serine proteases under similar in vitro conditions (Estévez et al. 2012; Ludewig et al. 2010).

Because Kunitz-type inhibitors require their native disulfide-bonded fold for high-affinity serine-protease inhibition, the dose-dependent inhibition of trypsin we observed up to complete inhibition at the highest dose supports correct disulfide pairing in recombinant arenin under non-reducing assay conditions. Overall, these results validate that the recombinant form of arenin retains the functional characteristics expected of a Kunitz-type serine protease inhibitor. Its ability to potently and specifically inhibit trypsin activity at low concentrations supports its structural and mechanistic classification within this protein family. This experimental confirmation reinforces the central hypothesis of this study, that recombinant arenin behaves as a canonical Kunitz-type inhibitor, and highlights its potential utility in biotechnological or therapeutic applications requiring precise regulation of serine protease activity.

### In vitro cytotoxicity and antitumoral activity

**Fig. 5:**
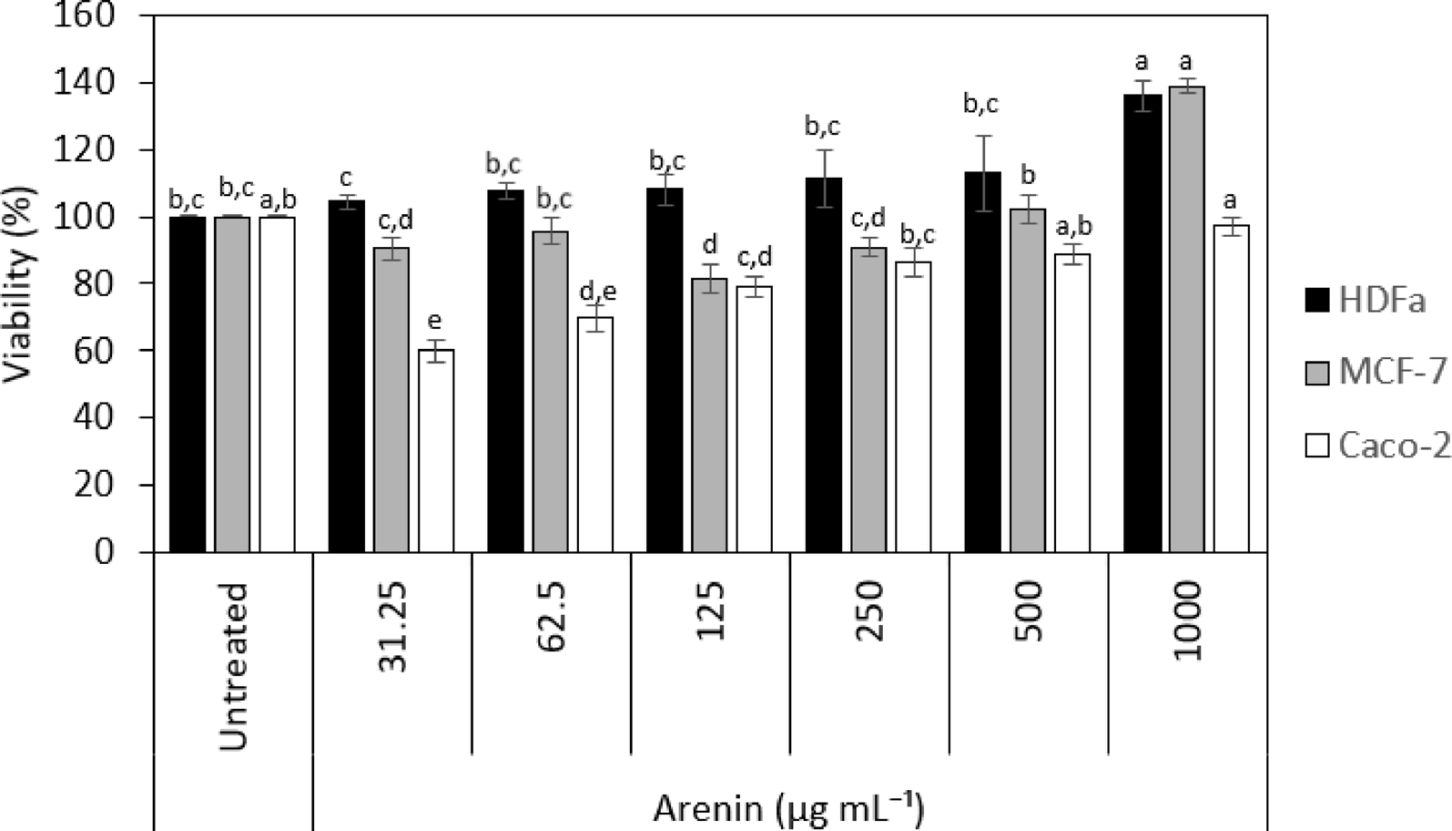
Viability of human dermal fibroblasts (HDFa), breast adenocarcinoma cells (MCF-7), and colorectal adenocarcinoma cells (Caco-2) after 48 h exposure to arenin. Data are expressed as the percentage of MTS-formazan absorbance (490 nm) relative to untreated controls (mean ± SD, *n* = 3). Statistical analysis was performed with one-way ANOVA followed by Tukey’s multiple-comparison test; Different letters ^abcde^ indicate statistical differences analyzed using the Tukey test (*p* < 0.05)

Human dermal fibroblasts (HDFa) responded to arenin with a strictly pro-survival profile. Viability climbed from 104.26% ± 2.08 at 31.25 µg mL^−1^ to a maximum of 135.93% ± 4.46 at 1000 µg mL^−1^ (Fig. 5**)**. Comparable mitogenic or cytoprotective effects have been reported for several amphibian-skin peptides that accelerate fibroblast proliferation and tissue repair, supporting the notion that arenin may activate regenerative signalling pathways such as ERK1/2 or PI3K/Akt in normal stromal cells (Yin et al. 2023). Stimulation of primary fibroblasts by Kunitz-type inhibitors has also been documented in plant orthologues, which enhance collagen deposition and wound closure (Bonturi et al. 2022).

MCF-7 (ER⁺) breast cancer cells exhibited a biphasic (hormetic) response to arenin. At 31.25 µg mL^−1^, viability fell modestly to 90.36% ± 3.33—a decrease that did not reach statistical significance versus control. Between 62.5 and 500 µg mL^−1^, cells partially recovered (viability 95.63% ± 3.90 to 102.34% ± 4.19; all *p* > 0.05). Strikingly, at 1000 µg mL^−1^ there was a pronounced proliferative burst (138.77% ± 2.28, *p* < 0.05). Estrogen-regulated serine proteases (PRSS23, matriptase, prostasin) drive MCF-7 proliferation and can be paradoxically stabilized by Kunitz-domain ligands (Chan et al. 2012; Martin and List 2019). Therefore, may be speculated that low arenin doses partially antagonize protease–receptor crosstalk, whereas high doses stabilize pro-growth complexes or engage compensatory mitogenic loops—analogous to the dose-dependent switches reported for the tick-derived Kunitz inhibitor Amblyomin X in ER⁺ tumors (Maria et al. 2018; Schmidt et al. 2020). This interpretation remains speculative, as no molecular assays were performed in this study to directly assess the signaling pathways involved. Future work will include targeted analyses (e.g., ERK1/2 or PI3K/Akt activation, PRSS23/matriptase activity) to validate the mechanistic basis of this hormetic response.

Caco-2 (hormone-independent) colorectal cancer cells proved highly sensitive at low and mid arenin concentrations. Viability dropped to 60.20% ± 3.40 at 31.25 µg mL^−1^ and remained significantly reduced through 250 µg mL^−1^ (*p* < 0.05), only returning to near-control levels at 1000 µg mL^−1^ (96.97% ± 2.48, *p* > 0.05). Caco-2 cells overexpress CD26/DPP4 and other membrane-bound serine proteases linked to invasion and stemness (Vázquez-Iglesias et al. 2019). Potent Kunitz inhibitors such as Amblyomin X induce apoptosis in CD26-rich carcinoma models by blocking proteasome-dependent survival pathways (Maria et al. 2018). Thus, the steep viability declines at ≤ 250 µg mL^−1^ is consistent with arenin targeting protease-driven survival mechanisms, while the rebound at 1000 µg mL^−1^ may reflect peptide aggregation or off-target binding that limits intracellular delivery.

Tukey’s HSD post hoc (letters in Fig. 5) confirms that in HDFa fibroblasts and MCF-7 cells, only the 1000 µg mL^−1^ dose differs significantly from untreated controls (*p* < 0.05), whereas all lower doses (31.25–500 µg mL^−1^) are statistically indistinguishable (*p* > 0.05). In Caco-2 cells, the control clusters with 1000 µg mL^−1^, while every dose ≤ 500 µg mL^−1^ produces a significant viability reduction (*p* < 0.05).

In summary, arenin enhances fibroblast proliferation and induces a dose-dependent hormetic profile in ER⁺ MCF-7 cells. In Caco-2 (hormone-independent) cells, arenin produced marked cytotoxicity at low-to-mid concentrations (viability dropped to 60.2% at 31.25 µg mL^−1^ and remained below 90% up to 250 µg mL^−1^), whereas viability recovered to near-control levels at higher doses (500–1000 µg mL^−1^; *p* > 0.05). This biphasic pattern suggests that protease-targeting effects predominate at lower arenin concentrations, while peptide aggregation or off-target interactions may mitigate cytotoxicity at higher concentrations. Although the contrasting behavior in normal HDFa fibroblasts versus carcinoma lines might seem counter-intuitive, comparable context-dependent responses have been documented for other Kunitz-type inhibitors—such as bovine pancreatic trypsin inhibitor (BPTI), tissue factor pathway inhibitor-2 (TFPI-2), and sialostatin L—which stimulate wound healing or angiogenesis in stromal cells yet suppress proliferation or invasion in breast, colon, and melanoma models. Analogous dual actions have also been observed with non-Kunitz peptides like human α-defensins. Thus, the selective activity of arenin fits within an emerging paradigm in which protease-directed peptides exploit divergent extracellular protease landscapes to spare healthy tissue while targeting malignant phenotypes (Hancock et al. 2016; Kobayashi et al. 2025; Lavergne et al. 2021; Mookherjee et al. 2020).

### Wound healing assay

The scratch assay was conducted to evaluate the regenerative potential of arenin under two stress conditions: serum deprivation (FBS-) and high glucose (HG), both of which are known to impair dermal fibroblast migration and delay wound healing (C. Liu et al. 2025; Shabestani Monfared et al. 2020). Recombinant arenin was tested at concentrations ranging from 31.25 to 1000 µg mL^−1^, and *Centella asiatica* (CA 10 µg mL^−1^), a phytotherapeutic well documented to accelerate fibroblast migration, was used as a positive control (Wang et al. 2024). A negative control group (C-) consisted of untreated cells. Wound closure (%) was quantified at 24, 48, and 72 h post-injury using FIJI image analysis.

#### FBS(−) condition

**Fig. 6:**
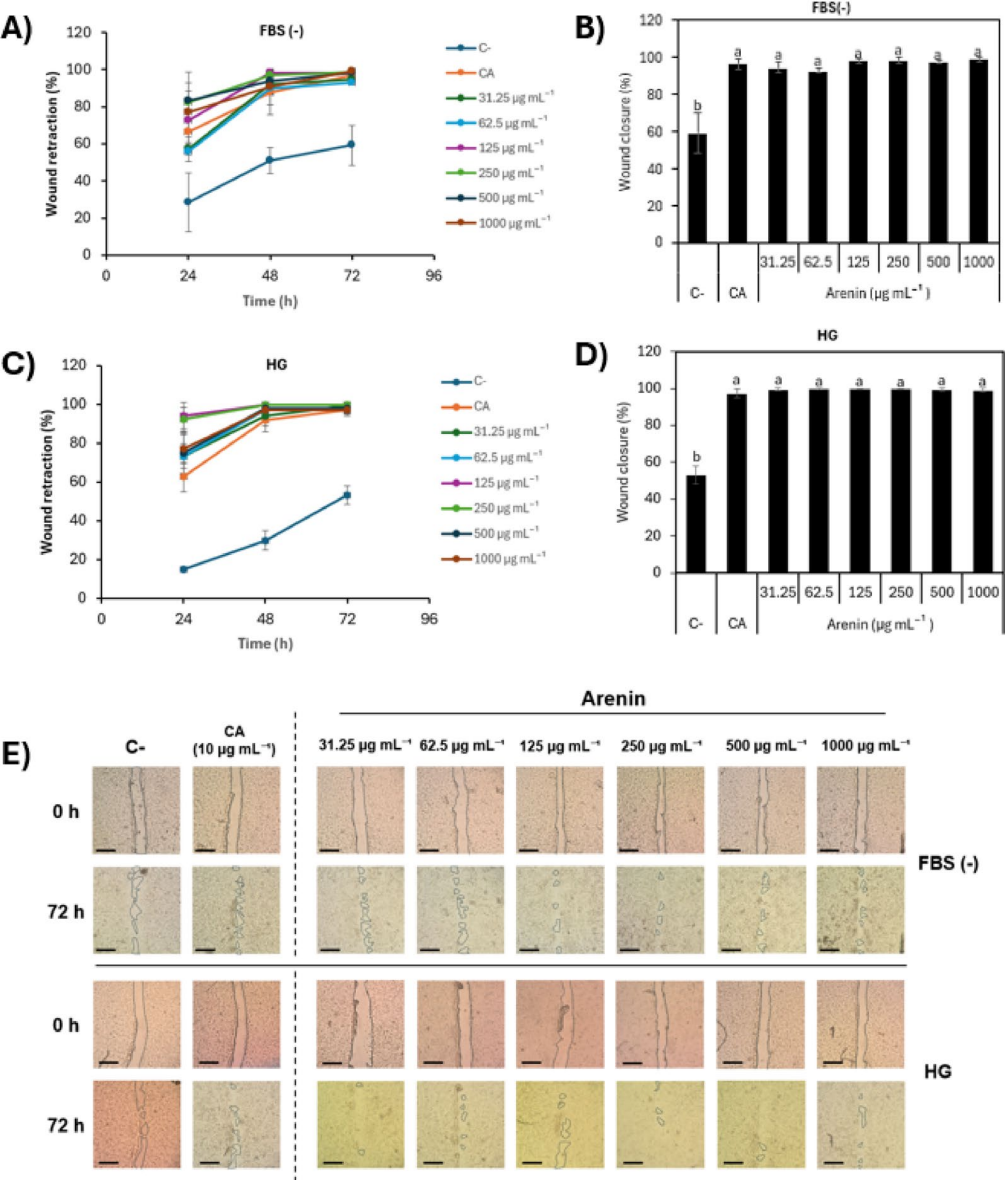
Effect of arenin on HDFa cell migration under high glucose (HG) and serum-deprived (FBS–) conditions. Scratch wound healing assays were performed in monolayers treated with arenin (31.25–1000 µg mL^−1^), *Centella asiatica* extract (CA, 10 µg mL^−1^), or left untreated (C–). **A**, **C** Time course of wound retraction (%) under FBS– and HG conditions at 24, 48, and 72 h. **B**, **D** Wound closure (%) after 72 h in FBS– and HG conditions. **E** Phase contrast micrographs at 0 and 72 h under FBS– (upper) and HG (lower) conditions show C–, CA, and arenin treatments. Dashed lines separate controls from arenin. Complete closure is observed in arenin- and CA-treated cultures, while sizeable gaps persist in C–. Bars/data points = mean ± SD (*n* = 3). One-way ANOVA with Tukey’s post hoc test; groups sharing the same lowercase letter are not significantly different (*p* > 0.05). Scale bars = 200 µm

Under serum-free conditions (Fig. 6A), the negative control exhibited a delayed wound closure, with only 28.33 ± 15.76% at 24 h, 50.97 ± 7.22% at 48 h, and 59.22 ± 10.86% at 72 h. In contrast, CA significantly accelerated wound healing, reaching 66.49 ± 6.67% at 24 h, 87.68 ± 11.87% at 48 h, and 96.90 ± 2.92% at 72 h, consistent with its reported stimulation of collagen synthesis and migration signalling (Wang et al. 2024). All arenin-treated groups showed improved healing compared to the negative control. For instance, 250 µg mL^−1^, arenin achieved 82.34 ± 10.39% at 24 h, 97.01 ± 3.56% at 48 h, and 98.61 ± 3.09% at 72 h.

At the end of the assay (72 h), all arenin concentrations and CA showed wound closure values above 94%, indicating significant regenerative activity (Fig. 6B). Statistically, all treatments were significantly different from the negative control (*p* < 0.05), but no differences were observed among treatments and CA, suggesting that the arenin effect is comparable to the effect of CA. These findings are visually supported by microscopy images at 72 h (Fig. 6E), where complete wound retraction is evident in treated groups but not in the control.

The rapid restitution under nutrient deprivation suggests arenin activates migration-centric pathways analogous to those mobilized by amphibian skin peptides such as tylotoin and cathelicidin-RL (Yin et al. 2023). Mechanistically, arenin may (i) stabilize actin polymerization waves that drive lamellipodial extension (Ahangar et al. 2022), (ii) up-regulate integrin α_5_β_1_ recycling to the leading edge (Schnittert et al. 2018), and (iii) increase MMP-9 secretion to remodel provisional extracellular matrix (Nishikai-Yan Shen et al. 2021). Such activities have been described for other Kunitz-domain proteins, including the hepatocyte-growth-factor activator inhibitor (HAI-1), which modulates pericellular proteolysis during tissue repair (Shimomura et al. 1997), and several plant Kunitz inhibitors that stimulate fibroblast proliferation in vitro (Bonturi et al. 2022).

#### High glucose condition

In the high glucose condition (HG) (Fig. 6C), the untreated control group again showed reduced healing capacity, with 15.0 ± 1.2% wound closure at 24 h, 30.0 ± 5.0% at 48 h, and only 53.0 ± 4.8% at 72 h, consistent with the literature reporting that hyperglycemia impairs fibroblast mobility and collagen deposition (Liu et al. 2025).

Treatment with arenin significantly improved healing under HG. The group treated with 250 µg mL^−1^, showed 93.0 ± 6.1% at 24 h, 100.0 ± 0.1% at 48 h, and 100.0 ± 0.2% at 72 h. Even the lowest concentration (31.25 µg mL^−1^) achieved complete closure at 72. Indicating a potent dose-independent response (Fig. 6D). All arenin groups and CA were superior to C- (*p* < 0.05) yet statistically equivalent among themselves, indicating a plateau in the dose–response curve, an effect also observed for peptide-based hydrogels that saturate pro-migratory signalling in diabetic models (Lu et al. 2025).

Across both stress paradigms serum deprivation and hyper-glycaemia arenin drove near-complete restitution of the scratch gap within 72 h, achieving 98.61 ± 3.09% closure under FBS- and 100.0 ± 0.20% under HG, marginally surpassing *Centella asiatica* (96.90 ± 2.92%); because this ceiling was reached across a 32-fold dose range (31.25–1000 µg mL^−1^), it likely reflects saturation of the integrin/FAK-ERK (Focal Adhesion Kinase–Extracellular Signal-Regulated Kinase) cascade and MMP-2/-9-mediated matrix clearance (Matrix Metalloproteinase-2/Matrix Metalloproteinase-9), a plateau phenomenon previously reported for Kunitz inhibitors and amphibian cathelicidins (Bonturi et al. 2022; Li et al. 2023; Pang et al. 2023). Figure 6E corroborates the quantitative data, revealing confluent fibroblast carpets in every arenin-treated monolayer. In contrast, untreated controls retain wide acellular gaps. Similar results have been observed with OA-RD17 and Andersonin-W1, two frog peptides that close diabetic or oxidative-stress wounds via similar MAPK- (Mitogen-Activated Protein Kinase) and TLR4 (Toll-Like Receptor 4) -centred mediated signaling pathways (Li et al. 2023).

Mechanistically, arenin is probably stabilizing actin polymerization waves that propel lamellipodia (Chen et al. 2024), accelerating integrin β1 recycling and focal-adhesion signalling (Huang et al. 2011), and recruiting surface-bound MMP-9 to remodel the provisional matrix (Dayer and Stamenkovic 2015). Because dermal fibroblasts choreograph both matrix turnover and immune tone in chronic wounds (Cialdai et al. 2022; Liu et al. 2022) recovering their motility under nutrient and metabolic stress positions arenin as a compelling candidate for difficult-to-heal ulcers. To delineate its therapeutic ceiling and breadth, future work should map arenin-induced changes in MMP-2/-9 activity, integrin trafficking, cytokine profiles, and angiogenic indices, ideally deploying peptide-laden self-healing hydrogels that have shown strong pre-clinical performance in diabetic wounds (Chhillar and Jaiswal 2025; Lu et al. 2025).

## Conclusions

Arenin was transformed from a scarce natural product into a readily accessible biomolecule through two optimized bacterial production routes, yielding milligram quantities of ≥ 90% pure peptide. It fully inhibits trypsin and exhibits context-specific biological effects, such as promoting fibroblast migration under stress and reducing the viability of Caco-2 carcinoma cells. In MCF-7 cells, a proliferative response observed at high concentrations was initially speculated to represent a hormetic effect; however, this interpretation remains hypothesis-generating, as no molecular analyses were conducted to confirm the involvement of specific signaling pathways. These findings position arenin as a promising candidate for regenerative applications and a potential lead for oncology research, pending optimization of delivery systems and further mechanistic validation relative to standard chemotherapeutics such as 5-fluorouracil. Future work will include structural characterization using these complementary methods to deepen understanding of arenin’s conformation–function relationship.

## Data Availability

The data supporting the findings of this study will be provided upon reasonable request.

## Abbreviations

6 × His: Hexahistidine tag
BAEE: Nα-Benzoyl-L-arginine ethyl ester
BCA: Bicinchoninic acid assay
BPTI: Bovine pancreatic trypsin inhibitor
CA: *Centella asiatica* Extract
Caco-2: Human colorectal adenocarcinoma
C–: Negative control
DTT: Dithiothreitol
EL: Elution
ER⁺: Estrogen receptor positive
FAK: Focal adhesion kinase
FBS: Fetal bovine serum
FT: Flow-through
HDFa: Human dermal fibroblasts, adult
HG: High glucose
HRP: Horseradish peroxidase
IMAC: Immobilized metal affinity chromatography
IPTG: Isopropyl β-D-thiogalactopyranoside
MAPK: Mitogen-activated protein kinase
MCF-7: Human breast adenocarcinoma cell line
MMP-2/9: Matrix metalloproteinase-2/matrix metalloproteinase-9 MM—master mix
Ni-NTA: Nickel-nitrilotriacetic acid
OD_600_: Optical density at 600 nm
PBS: Phosphate-buffered saline
PF: Purification factor
PVDF: Polyvinylidene difluoride membrane
S.D.: Standard deviation
SKTI: Soybean Kunitz trypsin inhibitor
TEV: Tobacco etch virus
TFPI-2: Tissue factor pathway inhibitor-2
TLR4: Toll-like receptor 4
WC%: Wound closure percentage
